# Highly localized droplet clustering in shallow cumulus clouds

**DOI:** 10.1073/pnas.2602976123

**Published:** 2026-08-04

**Authors:** Birte Thiede, Michael L. Larsen, Freja Nordsiek, Oliver Schlenczek, Yewon Kim, Eberhard Bodenschatz, Gholamhossein Bagheri

**Affiliations:** ^a^ Laboratory for Fluid Physics, Pattern Formation and Biocomplexity, Max Planck Institute for Dynamics and Self-Organization, Göttingen 37077, Germany; ^b^ Department of Physics, University of Göttingen, Göttingen 37077, Germany; ^c^ Department of Physics and Astronomy, College of Charleston, Charleston, SC 29424; ^d^ Department of Physics, Michigan Technological University Houghton, MI 49931; ^e^ Department of Physics, Cornell University, Ithaca, NY 14853

**Keywords:** cloud microphysics, droplet clustering, clustering hotspots, spatial intermittency, cloud holography

## Abstract

The size and spatial clustering of water droplets in warm clouds influence cloud evolution and radiation properties and thus are of fundamental importance for the Earth’s energy balance and water cycle. Some proposed mechanisms for droplet growth and precipitation initiation in warm clouds require strong droplet clustering. Resolving such clustering on sub-cm scales remains a challenge. The most detailed observations, collected from aircraft, showed weak mm-scale droplet clustering averaged over kilometer scales in stratocumulus clouds. Here we present spatially resolved in-situ holographic measurements from a tethered aerostat, demonstrating that clustering in shallow cumulus clouds is significantly stronger and far more localized than previously thought. These findings challenge current cloud microphysics frameworks, with implications for precipitation initiation and cloud modeling.

## Significance

Rain initiation in clouds containing only liquid droplets, known as warm clouds, remains poorly understood. Using a fast airborne 3D imaging instrument deployed within such clouds, we identified highly localized regions (about one meter) in which droplets are significantly more likely to be separated by small distances (less than one millimeter) than expected from random distributions. These observations provide direct evidence of strong but localized small-scale droplet clustering in raining warm clouds. Our results underscore the importance of resolving small-scale cloud processes in current theory and modeling frameworks as droplet clustering directly influences droplet growth rates and with it rain initiation. Our findings have important implications for improving predictions of rainfall onset and intensity in warm-cloud precipitation.

Clouds play a key role in determining the global energy balance and regulating the climate system; understanding their formation and interactions with atmospheric circulations is essential for accurate climate simulations (1). Although typically studied and modeled at macroscopic scales, the underlying processes that affect cloud formation, evolution, lifetime, and overall radiative forcing are influenced by microphysics driven by interactions at scales of individual droplets (2, 3). These are strongly intertwined with the highly turbulent atmospheric flow (4) and entrainment (5, 6). The local environment of a cloud droplet (including the presence or absence of other nearby droplets and their corresponding influence on temperature and vapor fields) is expected to have a significant impact on microphysical processes (3, 7, 8). Turbulence not only leads to complex mixing and transport of aerosols, temperature, and vapor, but may also have a yet-to-be-fully-understood effect on the inertia-driven clustering and collision–coalescence of cloud droplets.

Our limited understanding of microphysical processes is exemplified by our inability to fully explain how rain initiates in warm clouds, which is an unsolved mystery that has puzzled scientists for many decades (3, 9–22). A significant challenge in this process is overcoming the so-called “bottleneck” in warm clouds, which occurs for droplet diameters of approximately 30 to 80 μm. During this stage, condensational growth slows down, and collision efficiencies are low. The question, therefore, is why the bottleneck is overcome, rapid droplet growth occurs, and rain is initiated, as observed. (see, e.g., ref. 7).

Efforts to characterize particle clustering in clouds have been ongoing for at least 30 y, often using in situ measurements of cloud particle spatial positions (23–29). Typically, observations were made with aircraft-mounted probes that detected particle sizes and arrival times within a narrow optical field-of-view, enabling the reconstruction of one-dimensional spatial positions based on airspeed. Inferring three-dimensional clustering from such quasi one-dimensional data required assumptions about statistical isotropy and stationarity (30, 31). Digital in-line holography instead reconstructs true volumetric droplet spatial positions and sizes. Early efforts date back decades (25, 32–34), but only recent advances in hardware and software enable high-resolution reconstructions. One prominent instrument, the Holographic Detector for Clouds (HOLODEC), reconstructs up to ∼10 cm^3^ cloud volumes and provides the most spatially resolved in situ picture of three-dimensional cloud microstructure to date (5, 35–39). Nevertheless, characterizing scale-dependent clustering has remained challenging due to the limited number of droplets per holographic volume. Only by combining data from hundreds of consecutive holograms taken while flying through clouds have researchers recently detected weak but statistically significant millimeter-scale clustering averaged over 30+ km long stretches of stratocumulus clouds at large spatial intervals (typically 30 m) (40). The reported clustering shows qualitative features consistent with theoretical predictions of turbulence-driven inertial clustering (40–45). Although quantitative comparison with theory remains challenging, the clustering magnitudes reported by Larsen et al. (40) appear too small to substantially enhance droplet collision rates.

Here, measurements from the Advanced Max Planck CloudKite (${\text{MPCK}}^{\text{+}}$) instrument (46–48) allow for a much deeper look. With its higher measurement cadence and a slower-moving measurement platform, the volume fraction of the cloud examined increases by nominally a factor of 450 (*Materials and Methods*). This ∼2.5 order of magnitude improvement allows for far better spatial localization within a cloud transect of the observed microphysical clustering, which in turn reveals hints to plausible underlying clustering mechanisms and provides a vital observational step forward toward characterizing particle clustering magnitude on the sub-mm scales widely thought to be needed to influence microphysical process rates (see, e.g., ref. 49).

## Quantifying Droplet Clustering in Clouds

An important and often used tool to quantify statistically droplet clustering in clouds is the three-dimensional radial distribution function (RDF, or g(r), see Eq. **4** in *Materialsand Methods*). The RDF is designed to directly measure deviations from perfect spatial randomness (Poisson distributions), where $g(r_{○})=1$ indicates no correlations at the scale $r_{○}$ and $g(r_{○})=1.3$, for example, means that particles separated by distances between $r_{○}$ and $\left(r_{○}+\;\text{d}r\right)$ are found 30% more often than expected for a uniform Poisson distribution with the same underlying particle density. In some cases, clustering is expressed as the pair-correlation function, η(r)=g(r)−1 (e.g. ref. 50); this is equivalent to the RDF, apart from an offset of unity. In the following, the RDF is calculated for different subsections of a horizontal cloud transect. The RDF calculated over a subsection centered at cloud spatial position $x_{c}$ of length Δx ranging from $x_{c}-\frac{\Delta x}{2}$ to $x_{c}+\frac{\Delta x}{2}$ is described as ${\left\langle g\left(r,x_{c}\right)\right\rangle }_{\Delta x}^{\#h}$, where #h denotes the number of holographic sample volumes averaged over. Due to a roughly constant horizontal sampling rate #h/Δx is approximately constant. The radial distribution function is only statistically meaningful when examining statistically homogeneous and stationary data (50–54). Caution in interpreting the RDFs is needed for nonhomogeneous and nonstationary situations (55). The analysis of Larsen et al. (40) was based on the assumption that hundreds to thousands of consecutive holographic volumes collected over tens of kilometers of cloud were statistically homogeneous and stationary. This implies that the RDF would be independent of the position $x_{c}$ within a cloud. In previous works, it was assumed that relatively constant observed drop number concentration and drop size distribution throughout the data justified this assumption. However, as demonstrated below, the assumption of statistical homogeneity and stationarity over such large scales does not hold when measurements are performed at higher spatial resolution, even though the observed droplet size distributions and number concentrations remain relatively constant.

## In-Situ Measurements of Clouds at Unprecedented Scales

**Fig. 1.**
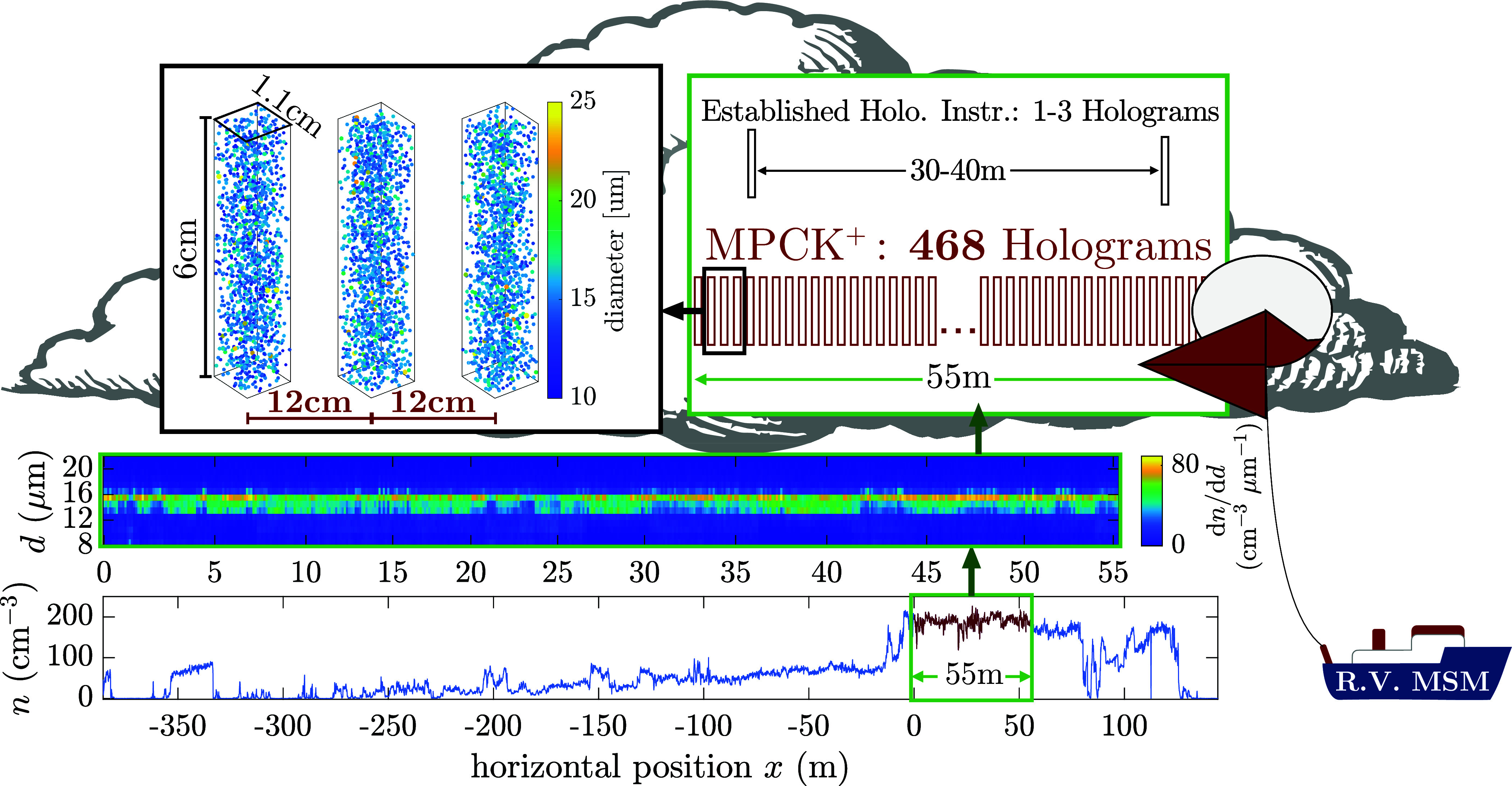
Direct cloud droplet measurements with the ${\text{MPCK}}^{\text{+}}$ during the ${\text{EUREC}}^{4}A$ campaign (48) launched from the Research Vessel Maria S. Merian (R. V. MSM, cruise number 89). Examples of three every 12 cm horizontally spaced holographically recorded droplets (positions and sizes) within a 1.1 cm × 1.1 cm × 6 cm volume are shown on the *Left Top*. The instrument box was operated below the CloudKite tethered balloon with a holographic imaging system running at 75 Hz at a TAS of 9 m/s (*Right*). The flight height was (847±20) m above sea level. The cloud base was at approximately 750 m and the cloud top at 1,200 to 1,800 m in the investigated region as observed by radar soundings from the research vessel (56). The droplet number concentration over 500 m of measurements is shown on the *Bottom*. For the marked 55 m of measurement the mean number droplet concentration as measured from the 468 holograms was around $200\;\;{\text{cm}}^{-3}$ and measurement height only varied by ±5 m. The droplet size distributions along the 55 m is unimodal with relatively minimal variability. *Top Right*, existing airplane-mounted holographic measurements (5, 40, 57) operating at ∼3 Hz and a TAS of ∼100 m/s would have only one to at most three measurement volumes in the same 55 m of cloud.

Here we report holographic measurements with the Advanced Max Planck CloudKite instrument box (${\text{MPCK}}^{\text{+}}$) instrument (46) inside shallow cumulus clouds during the ${\text{EUREC}}^{4}A$ campaign in the Atlantic trade wind regions near Barbados (48). As illustrated in Fig. 1, the high acquisition rate of the ${\text{MPCK}}^{\text{+}}$ holography system of 75 Hz, carried on the tethered helikite at a low true air speed (TAS) of ∼9 m/s, enabled highly resolved volumetric measurements of shallow cumulus cloud droplets with an interhologram distance of only 12 cm. This study analyzes the clustering signature in a segment of data collected within a precipitating shallow cumulus cloud. The selected data segment includes 468 consecutive holograms, covering a ∼55-m horizontal cloud region (Fig. 1. Following refs. 39 and 40, we focused on a cloud region with relatively stable droplet number concentration, liquid water content, and mass-weighted mean diameter. During this interval, the ${\text{MPCK}}^{\text{+}}$ altitude varied by less than 10 m. The turbulence dissipation rate in this cloud section was measured to be on average 0.03 m^2^/s^3^ and fluctuated between 0.009 and 0.164 m^2^/s^3^, the Taylor-scale turbulence Reynolds number was Reλ=1,600 and the integral length scale was on the order of 10m.

For consistency with earlier studies, the analysis was restricted to droplets with a minimum diameter of 10 μm within a 1.1 cm × 1.1 cm × 6.0 cm core region of the holograms. In this sample volume, a “superhologram” (combining droplets from all holograms) heat map showed no visual or statistically obvious deviations from uniform drop detection efficiency (*SI Appendix*). The droplet size distribution in the section was a constant narrow distribution with a mean of 14.9 μm throughout (Fig. 1), which is well above the 6 μm Nyquist detection limit of the holographic sensor. Additional details about the field study, instruments, measurement platforms, hologram postprocessing technique, and verification, and radial distribution function calculations can be found in methods and in *SI Appendix*.

## Evidence of Cloud-Droplet Clustering Averaged Over 55 m

**Fig. 2.**
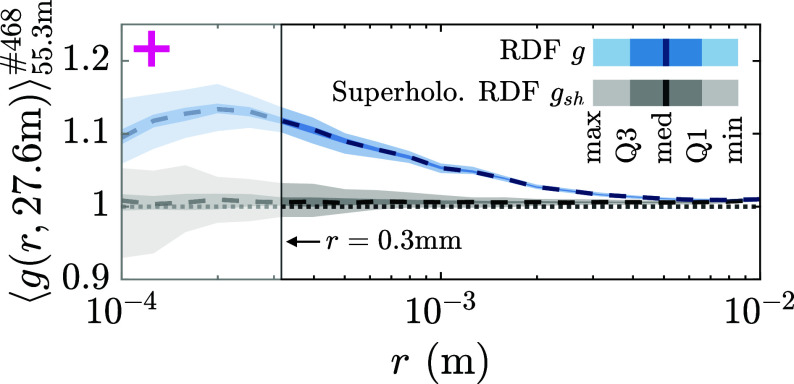
The radial distribution function averaged over the 468 holograms. Deviations from g(r)=1 can be due to both real, physical clustering and statistical/instrumental uncertainties; the gray line and shadings quantify some of these uncertainties. The gray bounds are calculated from artificial holograms, where observed hologram cloud droplet number variability is retained and droplet positions are randomly sampled from all droplet positions observed throughout the 55 m measurement interval (see *Materials and Methods* for more detail). The blue envelope shows g(r), the RDF from actual observed droplet position data perturbed by the depth position uncertainty ±150 μm. Due to measurement precision limitations, only r>0.3 mm is reliable; smaller distances are shown blurred. The regions where the two envelopes do not overlap (here on scales up to approximately 5 mm) show evidence for statistically significant droplet clustering throughout the interval. This approach, and the qualitative shape of this RDF curve, are largely consistent with the results published for marine stratocumulus clouds (40).

For calculating the three-dimensional RDF from the finite-volume holograms, earlier methods for calculating the RDF (58) were refined, rigorously tested, and validated using simulated datasets. The RDF for the entire 55-m domain is shown in Fig. 2, derived from all interparticle distances within the 7.26 cm^3^ volumes of the 468 holograms collected. As noted, values of g(r)>1 indicate a higher-than-perfectly-random frequency of interparticle distances at a given scale r, i.e. clustering at scale r. The uncertainty in g(r) may be influenced by multiple factors including sample size, measurement volume aspect ratio, droplet number concentration, and spatial scale r. Additionally, droplet data from any instrument have biases in detection and position accuracy. Thus, the RDF results require a meticulous interpretation with respect to measurement uncertainties, which is discussed below.

Fig. 2 shows two main traces: the blue line g(r) calculated from averaging over individual holograms in the 55 m domain and the gray line $g_{\mathrm{sh}}\left(r\right)$ from randomly drawing from pooling droplet data from all holograms, i.e. sampling from a superhologram generated from the associated 468 hologram interval. Ideal, unbiased measurements would yield $g_{\mathrm{sh}}\left(r\right)=1.0$ at all scales, but here minor deviations appear due to finite sampling and/or instrumental sensitivity limitations. To account for uncertainties in reconstructed droplet positions, g(r) and $g_{\mathrm{sh}}\left(r\right)$ were calculated for 100 resamplings, with positions varied within quantified uncertainties (59), e.g. ±150 μm along the camera axis. Solid lines show the median of these resamplings, and shaded areas span the minimum, maximum, and interquartile ranges for the 100-member ensemble. Given positional inaccuracies on the order of $10^{-4}$ m, retrieval of RDF statistics for spatial scales less than 0.3 mm cannot be reliably retrieved from this dataset.

## Highly Localized, Intense Clustering Hotspots Revealed

Consistent with the argument given in Larsen et al. (40), we argue there is evidence for droplet clustering if the envelope corresponding to the actual data g(r) lies outside the envelope corresponding to the superhologram $g_{\mathrm{sh}}\left(r\right)$. The width of the envelopes at each scale helps indicate the magnitude of the uncertainty of the radial distribution function. If at $r_{○}$ the RDF-envelope $g\left(r_{○}\right)=1+\left(\gamma \pm \delta \gamma \right)$ and the superhologram-RDF-envelope $g_{\mathrm{sh}}\left(r_{○}\right)=1+\left(\Gamma \pm \delta \Gamma \right)$, then the value of the actual RDF $g(r_{○})$ for the measured droplets can be estimated to be $g\left(r_{○}\right)∼1+\left(\gamma -\Gamma \right)\pm \left(\delta \gamma +\delta \Gamma \right)$.

**Fig. 3.**
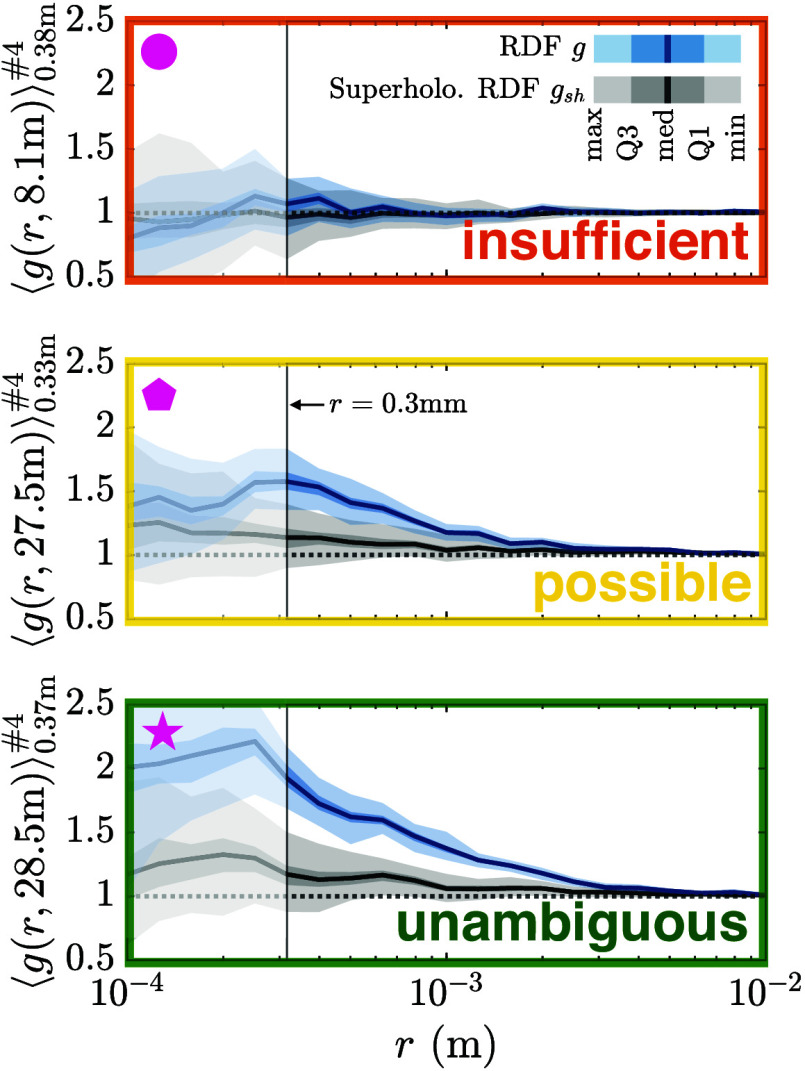
Examples of RDF-envelopes based on 4 consecutive holograms each to showcase classification of RDF results into categories “insufficient”, “possible”, and “unambiguous.” As described in Fig. 2, the RDF of measured droplets are shown with a blue envelope whereas the gray envelope serves as a reference revealing artifacts from sampling uncertainties and possible instrumental anomalies. For regions where g(r) and $g_{\mathrm{sh}}\left(r\right)$ envelopes fully overlap the clustering evidence is classified as “insufficient”, if separation only occurs for a few r values we classify clustering as “possible”, if, however, they separate for at least 4 different r values we classify the evidence for clustering as “unambiguous”.

The results shown in Fig. 2 qualitatively agree with the major findings of Larsen et al. (40); RDFs show net spatial clustering (the blue envelope lies above the gray), decrease monotonically with increasing spatial scale, and exhibit clustering near the turbulence dissipation range. Quantitatively, however, the clustering observed here is about 5 to 10 times stronger than reported in Larsen et al. (40). The difference in the magnitude of the clustering signature is surprising at first sight; is this due to different cloud types (marine shallow cumulus vs. marine stratocumulus) and/or perhaps due to more intense turbulence? Could it be that the distance of 30 m between measurements in Larsen et al. (40) averaged out the observed clustering and that clustering appears stronger if analyzed on Δx=55m compared to Δx≈O(km)? Thanks to the very high spatial sampling of only 12 cm distance between holograms by the ${\text{MPCK}}^{\text{+}}$ we can probe the RDF at smaller averaging scales, i.e. subdividing the interval into very fine domains and show the importance of Δx in analyzing droplet clustering. This is possible here due to the sufficient number of drops per hologram due to the large sample volume and high droplet concentration. Fig. 3 illustrates this approach using subdomains of four consecutive holograms taken at locations within the 468-hologram (55 m) domain. These 4-hologram subdomains average only over ≲0.4 m of cloud. Each subplot compares the measured g(r) envelope (blue) with the associated superhologram $g_{\mathrm{sh}}\left(r\right)$ envelope (gray). Compared to the full-domain envelopes in Fig. 2, these smaller subdomains show substantially wider envelopes, indicating the higher uncertainty resulting from sampling fewer droplets. Unlike in Larsen et al. (40), however, the parameters of these data do not require thousands of consecutive holograms and correspondingly kilometers-long scales to reveal enough statistical evidence to argue for unambiguous presence of clustering; some of these ∼0.5 m scales show unambiguous evidence for droplet clustering. We use the range of scales in which the measured RDF g is outside of the uncertainty envelope of the superhologram RDF $g_{\mathrm{sh}}$ to classify the clustering evidence of each 4-hologram RDF as either “insufficient,” “possible,” or “unambiguous.” Examples of an RDF result for a 4-hologram subsection with different clustering evidence are each shown in Fig. 3. For instance, although g(r=0.3mm)≈1.1 in the *Top* panel is comparable to the full-domain value in Fig. 2—where clustering evidence was found—the increased uncertainty in this 4-hologram subset (demonstrated by the thickness of the associated envelopes, caused by statistical uncertainties due to fewer droplet pairs at small r) makes it a statistical overreach to draw the same conclusion here. Thus, the classification insufficient was chosen instead of “no clustering;” there may indeed be clustering, but the limited droplet count precludes confident determination. The middle subplot shows g(r) and $g_{\mathrm{sh}}\left(r\right)$ for another 4-hologram sample (further into the region when $x_{c}=27.5$m instead of $x_{c}=8.1$m), where the envelopes do not overlap in a small region near r=0.4mm, and clustering is categorized as “possible.” In contrast, the lower subplot of a different 4 hologram sample reveals a clear separation of envelopes over a significant range of scales (between 0.25 and 3 mm) and clustering evidence is thus “unambiguous.”

**Fig. 4.**
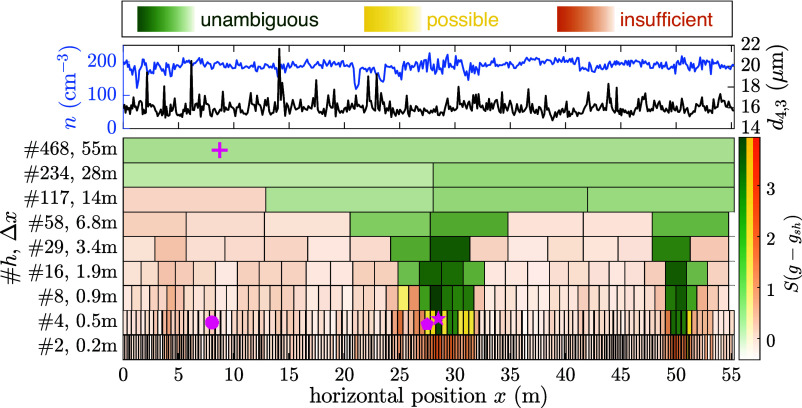
An overview of where clustering evidence can be found within subdomains of the ∼55 m data. The *Top* panel shows traces of the droplet number concentration n and the mass-weighted mean diameter $d_{4,3}$. The *Bottom* panel shows which of the three clustering evidence classes (see Fig. 3 for reference) each subinterval is classified into. Green indicates unambiguous evidence of statistically significant droplet clustering. The marked subintervals (plus, circle, pentagon, and star) correspond to the shown RDFs in Figs. 2 and 3. The color opacity show a measure of RDF amplitude, computed by the sum of the difference of g(r) and $g_{\mathrm{sh}}\left(r\right)$ across scales, i.e. $S\left(g-g_{\mathrm{sh}}\right)=\sum_{r\in \{0.3\;\mathrm{mm},\cdots ,10\;\mathrm{mm}\}}[\mathrm{med}\left(g\left(r\right)\right)-\mathrm{med}\left(g_{\mathrm{sh}}\left(r\right)\right)]$, where “med” stands for the median. $S(g-g_{\mathrm{sh}})$ is a crude measure of the RDF amplitude above the uncertainty level. Note that some of the strongest RDF amplitudes seem to occur even where there is “insufficient” evidence for clustering; with 2 holograms, the differences between the superhologram-RDF that make up $g_{\mathrm{sh}}\left(r\right)$ cannot be reliably distinguished from the measured g(r) values.

By using this classification of clustering evidence based on g(r) and $g_{\mathrm{sh}}\left(r\right)$ across disjoint subintervals Δx of the 55-m transect, our findings reveal that the clustering is far more localized than previously anticipated. Fig. 4 highlights (in color) the subintervals where clustering is evident; the modest clustering signal over the entire 55-m domain shown in Fig. 2 evidently results from stronger, localized clustering in only a small fraction of the domain. The spatial extent of the clustered region is no more than a few meters in horizontal extent within these shallow cumulus clouds. What initially erroneously appears as “weak ubiquitous” clustering over the full domain can now be localized and properly attributed as “stronger but intermittent” clustering that manifests on meter-scale subdomains of the cloud region. The measurement and statistical uncertainty limits us to averaging of 50 to 100 cm horizontal extent (4 to 8 hologram subdomains) to find a clear clustering signal, which also allows using $g_{\mathrm{sh}}$ as measurement reference. At this point, the exact spatial extent of the clustering hotspots can therefore not be measured. Clustering evidence at a given cloud location $x_{c}$ and averaging scale Δx points to localized subscales with stronger clustering signatures (i.e., no “green” shading lies below an “orange” shading in Fig. 4). These observations, which align with additional ${\text{MPCK}}^{\text{+}}$ data from ${\text{EUREC}}^{4}A$ (see e.g. *SI Appendix*, Fig. S1), reinforce that while small-scale clustering may seem pervasive in large domains as in Larsen et al. (40), it is likely often strongly localized, with its true magnitude hidden by the required averaging in prior aircraft measurements. The strong effect of dilution or hiding of the true clustering signal when averaging over too large scales is exemplified in this paper: Even just averaging over this full Δx=55m (compared to kilometers in ref. 40) reduced the value of $g\left(0.3\;\;\text{mm}\right)-g_{\mathrm{sh}}\left(0.3\;\;\text{mm}\right)$ by about an order of magnitude compared to calculating the RDF on Δx=0.5m (#h=4 hologram) scales (compare Fig. 2 with the *Bottom* panel of Fig. 3).

## Discussion and Conclusions

The in-situ observations presented here show that cloud droplet clustering is highly intermittent. Most of the sampled cloud does not exhibit statistically significant clustering, whereas clustering, when present, is confined to localized meter-scale hotspots with strong amplitudes. This contrasts with previous observations in stratocumulus clouds, where clustering was found to be weak and ubiquitous (40). We attribute this discrepancy primarily to the high-cadence measurements enabled here by ${\text{MPCK}}^{\text{+}}$, which resolve and preserve localized clustering signals in the RDF rather than averaging them out.

The identified clustering hotspots show no clear correlation with variations in droplet number concentration or mass-weighted mean droplet diameter (*Top* panel of Fig. 4). Within the present dataset, these conventional microphysical variables therefore do not provide a discernible indicator of where enhanced clustering occurs. This suggests that hotspot occurrence is not simply controlled by bulk droplet properties, but may depend on unresolved local dynamical variability or other fine-scale environmental factors.

Turbulence is likely an important contributor to the observed clustering, especially given the localized nature of the signal. The likely contribution of turbulence is supported with extensive laboratory, theoretical, and modeling work showing that turbulence can induce clustering of inertial particles, modify relative droplet velocities, and affect collision statistics (e.g. refs. 7, 8, 15, 22, 60–70, and references there in). However, the present study does not provide a definitive attribution of the clustering mechanism. It is a compelling opportunity, to investigate the potential role of turbulence, among other factors, using the ${\text{MPCK}}^{\text{+}}$ PIV system, which enables characterization of localized in-cloud turbulence at unprecedented spatial resolution in future studies.

Overall, these observations demonstrate that cloud droplet spatial distributions can contain strong localized structures that are not apparent from bulk microphysical quantities alone. The RDFs presented here probe scales of r>0.3m rather than near-contact droplet separations and therefore cannot directly quantify collision enhancement or coalescence rates. Nevertheless, the observed intermittency in the precipitating cloud is qualitatively consistent with the idea that rare, localized microphysical events may contribute disproportionately to warm-rain initiation (18). These findings highlight the importance of intermittent, localized processes in cloud microphysics and establish a pathway for investigating their relevance to parameterizations used in weather and climate models.

## Materials and Methods

### Measurements: The Field Campaign and Instruments.

The holographic measurements used in this study were collected during the ${\text{EUREC}}^{4}A$ campaign (48) using The Max Planck CloudKite (MPCK) operated aboard *R.V. Maria S. Merian* (cruise number 89). This campaign endeavored to measure properties of shallow cumulus clouds over the North-Atlantic near the coast of Barbados. The Max Planck CloudKite is an atmospheric research platform that integrates a tethered lighter-than-air aerostat, a 250 m^3^ helikite, operated by a large gasoline winch with a maximum flight height of 1.4 km during ${\text{EUREC}}^{4}A$ campaign carrying the Instrument Box ${\text{MPCK}}^{\text{+}}$ (48).

The ${\text{MPCK}}^{\text{+}}$ instrument box is described in detail in ref. 46. It is equipped …” to “The ${\text{MPCK}}^{\text{+}}$ instrument box is described in detail in ref. 46. It is equipped with sensors for measuring relative humidity, temperature and pressure, pitot tubes for one- and three-dimensional true airspeed measurements, an inertial navigation system combined with GPS, a Fast Cloud Droplet Probe (FCDP, SPEC Inc.), and most importantly two custom-built imaging systems: namely particle image velocimetry (PIV) and holography (developed at MPI-DS) systems with overlapping sample volumes. The PIV system provides localized turbulence characteristics, such as the dissipation rate, within a 13 cm × 9 cm field of view, using a 4 mm thick green (532 nm wavelength) laser sheet, a thin, planar beam of laser light that illuminates particles only within the measurement plane, allowing their motion to be tracked in two dimensions. It operates at 15 Hz, capturing velocity fields at high spatial resolution. The holographic imaging system features a 5,120×5,120 pixel sensor with an effective 3 μm pixel size, illuminated by a 355 nm laser at 75 Hz. It reconstructs droplet size and position within a 1.1 cm ×1.1 cm× 6 cm core volume (a total net probing volume of 0.54 L/s), with a full probing range extending to 15 mm ×15 mm× 21.9 cm. Both imaging systems are synchronized, ensuring that every fifth double-frame PIV image aligns with a hologram captured precisely in between. The tethered-balloon nature of the platform enabled data collection at a true airspeed of about 9 m/s, significantly reducing the spatial gap between successive holograms down to about 12 cm.

The data analyzed in this manuscript were collected on 12 February 2020 at 22:04 UTC at 13.2524°N, 57.3908°W, with altitudes ranging from 842.1 m to 852.1 m above mean sea level. Cloud radar data operated aboard the R.V. MSM89 (56) indicate a cloud base at approximately 0.75 km, with the cloud top ranging between 1.2 and 1.8 km during the time interval investigated here. The radar measurements and an on-board camera indicate precipitation. The size distribution is stable throughout the analyzed cloud section. It is a narrow unimodal distribution with a mode around 15 to 16 μm as shown in Fig. 1.

A summary about the ${\text{MPCK}}^{\text{+}}$ measurements used in this study and comparison to HOLODEC measurements used in ref. 40 is presented in Table 1.

**Table 1.** Comparison between the parameters of the datasets used in ref. 40 (HOLODEC) and this work (${\mathrm{MPCK}}^{+}$)

Instrument	HOLODEC	${\text{MPCK}}^{\text{+}}$
Measurement platform	NSF/NCAR GV HIAPER	Tethered kite
Field campaign	CSET (July 2015)	${\text{EUREC}}^{4}A$ (2020)
Measurement location	Eastern Pacific Ocean	Northern Atlantic Ocean
Cloud type	Marine stratocumulus	Shallow cumulus
Measurement platform translational velocity	∼100 m/s	∼9 m/s
Holographic volume used	0.6 cm × 0.6 cm × 10 cm	1.1 cm × 1.1 cm × 6.0 cm
Minimum retained cloud drop diameter	10 μm	10 μm
Spatial separation between samples Δx/#h	∼30 to 40 m	∼12 cm
Analyzed cloud droplets	[3.5 to 20.1]$\times 10^{5}$	6.4$\times 10^{5}$
Measured mean drop number concentration	[75.3 to 279.0] ${\text{cm}}^{-3}$	188.9 ${\text{cm}}^{-3}$
Cloud volume examined	[4.6 to 7.2]$\times 10^{-3}$ m^3^	3.4 $\times 10^{-3}$ m^3^
Mean diameter of studied droplets	[15.0 to 20.2] μm	14.9 μm

In ref. 40, there were 4 flight intervals presented, whereas this study presents only 1 flight interval so ranges are given for some fields in the HOLODEC information.

### Hologram Processing and Verification.

The hologram processing consists of several steps; first the raw holograms are background- and noise- filtered. Afterward the wavefront is reconstructed along the z-axis. A threshold is applied to the reconstructions to identify dark objects. These objects are classified with a Convolutional Neural Network (CNN, a machine-learning algorithm for image classification) and particles are identified and their z-focus, their size, and x−y-center is determined. The reconstruction is based on ref. 71 and the classification and verification of the processing, as well as the complete processing chain, is described in detail in ref. 59.

The cloud volume sampled by the ${\text{MPCK}}^{\text{+}}$ falls loosely into a rectilinear parallelepiped having dimensions of approximately 1.5 cm × 1.5 cm × 21.9 cm. The sensitivity of the instrument is not uniform over this domain, however, so the results presented here are constrained to be from a core of dimensions 1.1 cm × 1.1 cm × 6 cm, with the short dimensions centered on the middle of the field-of-view of the sensor, and the long dimension spanning from a short distance of 6 cm from the imaging plane out to 12 cm from the imaging plane. This particular subvolume was chosen because a global “heat-map” of particle detections showed visually uniform behavior over this volume when looking at a superhologram of all particle detections. We have also investigated the performance of the ${\text{MPCK}}^{\text{+}}$ holographic system thoroughly using the Cloud Target (59). The CloudTarget, which is a set of opaque circles of known size and position patterned on a chrome photomask, is a verification tool developed to experimentally quantify detection efficiency and assessing size and position errors. It provides a reliable ground truth for optimizing hologram processing parameters, including detection, sizing, and classification thresholds, and it facilitates evaluations of size-dependent detection efficiency and uncertainties. The CloudTarget verification shows that the ${\text{MPCK}}^{\text{+}}$ holographic system is associated with >90% detection for particles >10 μm diameter within the chosen subvolume. The idea of using data from a subselected core with uniform particle detection sensitivity is similar to the approach shown in figure 4 of ref. 58, but with the ${\text{MPCK}}^{\text{+}}$ system having reliable/uniform detection over a less extreme aspect ratio than the HOLODEC instrument.

Although we believe that the ${\text{MPCK}}^{\text{+}}$ instrument system may be capable of reliably detecting particles down to about 8 or 9 μm in diameter (59), we used the same minimum 10 μm diameter cloud droplet size used in ref. 40 to allow for a direct comparison.

In ref. 40, HOLODEC subvolumes measuring 0.6 cm × 0.6 cm × 10.0 cm were utilized, and the data were acquired on an airplane platform moving at nominally 100 m/s and images acquired at 3 Hz. With the flight direction aligning with one of the 0.6 cm sample dimensions. This means that only approximately 0.6 cm out of every 30 m along the flight direction was observed, or about $2\times 10^{-4}$ of the horizontal transect of the 0.6 cm × 10 cm cross-section of flight.

Here, using ${\text{MPCK}}^{\text{+}}$ subvolume parameters as outlined above, we find that approximately 1.1 cm out of every 12 cm, or about 0.09 of the horizontal transect of the 1.1 cm × 6 cm cross-section of the flight was used. Thus, this study not only acquires about 250 holograms in the same spatial interval where another prominent study (40) acquired 1, but the volume fraction of the cloud examined is 450 times greater. See Table 1 for other comparisons between the dataset used in ref. 40 and ${\text{MPCK}}^{\text{+}}$.

### Radial Distribution Function Calculation (RDF).

Efforts to use in situ measurements to estimate the RDF in clouds have been in progress for nearly two decades; the desire to estimate in-cloud RDFs were design considerations for the development of the HOLODEC (35–37).

Early HOLODEC datasets were explored with a specific goal to understand and study sub-cm 3-dimensional structures using a variety of different statistical approaches (5, 38, 39), but using HOLODEC data for direct in situ estimates of the RDF could not be done until algorithms were developed to estimate the radial distribution from convex experimental volumes with a variety of aspect ratios (58). Following this work, in situ RDFs from HOLODEC data could be estimated, but relied on averaging statistics from hundreds to thousands of consecutive holographic images corresponding to averaging the spatial statistics for regions spanning tens of kilometers (40). Despite the extensive required spatial averaging, previous work (40) was able to report the first in situ estimate of the RDF in (stratocumulus) clouds on mm-scales.

This work leans heavily on these previous efforts to characterize the instrumentation and develop the algorithms necessary to report RDFs with sufficient clarity. As alluded to in the main text (and discussed at some length in refs. 40 and 58, and in particular the supplemental materials of ref. 40), the process of assigning uncertainty bounds to in situ measurements of an RDF is complicated and nuanced. Previous work has laid an important foundation to understand and quantify these uncertainties, but in this study we made additional improvements that are outlined below.

Following many other authors (e.g., refs. 3, 40, 41, 50, 53, 58, 61, and 72–74), we have used the RDF to give a localized quantification of the scale-localized departures from perfect spatial randomness (a discrete Poisson distribution, e.g., ref. 26). This statistical tool (and/or the similarly related statistical tool called the pair-correlation function) is described in detail in many other places (e.g., refs. 3, 26, 50, 53, 55, 72, and 75) but, briefly, the RDF g(r) quantifies the scale-localized clustering present among particles in a three-dimensional, isotropic, statistically homogeneous medium.

Conceptually, g(r) reports the probabilistic enhancement of finding two particles separated by distances between r and r+dr. In a perfectly random three-dimensional volume V containing N total particles, the probability of finding a particle in an infinitesimal volume dV a distance r from a known particle can be found via:

[1] $$\begin{array}{c} p\left(dV\right)=\frac{\left(N-1\right)}{V}dV \end{array}$$

since the presence of the first particle has no bearing on the probability of detecting another particle in volume dV, and the differential volume is assumed small enough that the probability of more than one detection within the infinitesimal volume is negligible.

For a system with N total particles, then, the total expected number of particles having a “match” a distance between r and r+dr away can be computed by multiplying this probability by N:

[2] $$\begin{array}{c} \tilde{\psi }\left(r\right)=\frac{N(N-1)}{V}dV \end{array}$$

In practice, the number of particle pairs separated by distances between r and r+dr could be larger or smaller than this value; if we call this observed number of particle pairs ψ(r), then the ratio $\psi \left(r\right)/\tilde{\psi }\left(r\right)$ gives the “radial distribution function” g evaluated at scale r. If g(r)=1.3, for example, it means that there are 30% more particle pairs separated by scale r than would be expected in a perfectly random, statistically isotropic and homogeneous volume having the same total number of detected particles. Thus, we modify Eq. **1** to reflect the actual probability of finding a particle in a volume dV separated by r from a known particle as

[3] $$\begin{array}{c} p\left(dV_{r}\right)=\left[\frac{\left(N-1\right)}{V}dV_{r}\right]g\left(r\right), \end{array}$$

where the subscript r refers to the separation between the known particle location and the location of the infinitesimal volume.

Operationally, g(r) can be computed from an idealized dataset containing N total droplets in volume V by computing

[4] $$\begin{array}{c} g\left(r\right)=\sum_{j=1}^{N}\frac{\psi_{j}\left(r\right)/N}{\left(N-1\right)\left(\frac{dV_{r}}{V}\right)} \end{array}$$

with $\psi_{j}\left(r\right)$ counting the number of particles having their centers a distance between r and r+dr from the jth detected particle in the dataset, and $dV_{r}$ representing the volume of a spherical shell with inner and outer radii r and r+dr, respectively (58). This simplified formulation ignores some complications associated with measurement volume boundaries, but other than that gives an accurate summary of how g(r) can be computed from an experimental dataset.

## Supplementary Material

Appendix 01 (PDF)

## Data Availability

The holographic cloud droplet dataset used for the analysis is available via Zenodo: https://doi.org/10.5281/zenodo.21295056 (76).
