# Supplementary material for: Highly localized droplet clustering in shallow cumulus clouds
Source: Proc Natl Acad Sci U S A. 2026 Aug 4;123(32):e2602976123. doi: 10.1073/pnas.2602976123 (PMC13462724; doi:10.1073/pnas.2602976123)
Supplement: Supplementary file 1 [file pnas.2602976123.sapp.pdf]

PNAS

Supporting Information for

Highly Localized Droplet Clustering in Shallow Cumulus Clouds

Birte Thiede, Michael L. Larsen, Freja Nordsiek, Oliver Schlenczek, Yewon Kim, Eberhard Bodenschatz, Gholamhossein Bagheri

Gholamhossein Bagheri.
E-mail: gholamhossein.bagheri@ds.mpg.de

This PDF file includes:

Supporting text
Figs. S1 to S2
SI References

Supporting Information Text

A. The Utilized Algorithm for RDF calculations. When calculating the RDF for a convex finite spatial volume (all connections between points lie within the volume, like experimental holographic volumes used in this study), the commonly-used approach of using periodic boundary conditions to look for particle "pairs" near the edge of the measurement volume doesn't make physical sense (see, e.g., (1)). Under these conditions, many investigators use a "guard-area" approach (or "minus-sampling" approach) to calculate the number of particle pairs separated by distance r in the computation of the RDF. In this approach, the sample volume is broken into two disjoint pieces – (1) a central region where particle detections are always used, and (2) the measurement region outside this central region (called a guard-area) where particles are retained to allow for particle matching (2). In this formulation, the sum in equation 4 in the main manuscript is taken over particles in the central region only, whereas particle "pairs" can be found in both the central region and the guard area (so long as at least one member of the pair exists in the central region); the volume V is limited to the volume of the central region. This approach *does* work, but makes very inefficient use of measured data in volumes with non-unity aspect ratios, and still requires limiting both the central region *and* guard areas to measurement sub-domains where particle detections are reliable (1). Given the decrease of instrument sensitivity towards the edges in x-y direction, hence having to limit the effective sample volume already, and the aspect ratio of the MPCK$^+$ holographic system, using such an approach either profoundly limits the spatial scales where $g(r)$ can be reliably calculated and/or requires constraining the utilized spatial volume within the detector sampling area so far as to make use of the data rather ineffective. The method of computing the RDF in this work uses an altered version of the algorithm outlined in (1) with some slight modifications. Unlike in this previous study where a static look-up table was used to modify the "effective volume" $\mathrm{d}V_{r_{i,j}}$, the algorithm used in this study utilized an on-the-fly normalization described briefly as follows:

For each particle in the (trusted/utilized part of the) sample volume, for each radial distance of interest r_i, and for (fixed) shell thickness $\mathrm{d}r = 100\ \mu$m we find the expected number of particle pairs separated from each other by distances ranging from r_i to $r_i + \mathrm{d}r$ from $(N-1)\left(\frac{\mathrm{d}V_{r_i}}{V}\right)$ with N the total number of particles in the (used part of the) holographic sample volume, V the volume of the (used part of the) holographic sample volume, and $\mathrm{d}V_{r_i}$ the estimated volume of a spherical shell having inner radius r_i and outer radius $r_i + \mathrm{d}r$ centered on the particle *that still lies within the used part of the sample volume of the hologram*. In (1) this was done by using a gridded look-up table and then finding the closest entry of the actual particle position to a pre-assigned array of possible particle positions. This is completely acceptable and validated in (1), but requires a different look-up table for each possible instrument sample volume or aspect ratio. Here, instead, we place 1000 random particles spatially uniformly randomly in the specified spherical shell and count how many of them lie within the retained sample volume. We then take the fraction still within the sample volume and multiply it by the volume of the full spherical shell to estimate $\mathrm{d}V_{r_i}$. Tests of this new algorithm that exactly mimic those presented in (1) were carried out; this algorithm gives consistent results with expected $g(r)$ curves for Poisson simulations and Matérn Cluster processes embedded in box-shaped volumes with various different aspect ratios.

B. Averaging over Intervals. . In (3) it was shown that for a single HOLODEC volume with 70 detected drops per cubic centimeter the retrieved RDF for a collection of particles that are known to be perfectly randomly distributed can vary from about 0.7 to 1.5 at mm-scales. In this case the deviation from unity is due purely to counting statistics and this extreme spread is the reason that so many consecutive holographic images had to be combined to reveal the weak mm-scale clustering signatures seen in that study.

The way clustering statistics from consecutive holograms were combined in (3) was quite simple; since the drop number concentration was approximately constant throughout each analyzed flight interval, the reported $g(r)$ for an interval containing N_{hol} consecutive holograms was computed by taking an average of the individual (noisy) RDFs for each hologram $g_i(r)$, e.g.:

$$g(r) = \frac{1}{N_{\mathrm{hol}}} \sum_{i=1}^{N_{\mathrm{hol}}} g_i(r) \qquad [1]$$

The goal, however, is to use all of the data from the observed volume of cloud to estimate the (presumably physically constant) $g(r)$ for the entire domain. As such, mere arithmetic averaging of the $g_i(r)$ for all holograms in the sample is not an unbiased estimator of $g(r)$. Formally, this is a consequence of Jensen's inequality (4, 5).

Our revised computational approach makes the subtle change to record each variable that goes into the expression in equation 4 in the main manuscript separately so that the full-interval value for $g(r)$ can be more accurately estimated. This modification was validated using the same test-cases used in (1).

C. RDF Uncertainty. Calculating the uncertainty for an RDF is nuanced; it is a derived quantity, arising from ratios of measured and inferred properties. As such, putting on traditional error-bars without explanation can be easily misinterpreted (6). In previous work, it has generally been easier to demonstrate the intertwined nuances of statistical, measurement, and sampling uncertainties via use of carefully designed numerical simulations. In this work, we use similar approaches – preferring to show envelopes instead of lines with error bars. In each case, the light shading is associated with showing the full range of observed $g(r)$ values from an ensemble of 100 simulations that replicate, to the best extent possible, the known limitations, biases, and uncertainties associated with the actual measurement; the darker shading shows the distance between the 1st and 3rd quartiles,

and the dotted lines indicate the median simulation value. Explanations on how a single set of observations can result in an ensemble of values is explained more extensively below.

Depth Latching / Jitter. The data set from the MPCK$^+$ system (and the HOLODEC system, for that matter) isn't isotropic; the algorithm that is used to take the holographic images and reconstruct the particle positions has different precision in the horizontal and depth (vertical) dimensions. Much like the HOLODEC system, the MPCK$^+$ particle position reconstructions are "gridded" along the z (depth) direction to the nearest 100 μm plane. This suggests that the particle positioning along this direction has an uncertainty of at least $\pm$ 50 μm, even if the instrument and processing algorithms operate perfectly. Because of this, we hesitate to show RDF calculations for any spatial scale at or near 100 microns, since these distances are at least comparable to the uncertainty in particle placements. Laboratory testing of the MPCK$^+$ with the CloudTarget suggests that, in practice, algorithmic reconstruction along the depth dimension is likely accurate to nominally $\pm$150 μm.

To account for any affect that this depth-uncertainty has in calculating the RDF, all plots of $g(r)$ presented are shown not as a line, but rather as an envelope. To generate this envelope, we resampled each droplet distribution 100 times – each time "jittering" the z-coordinate of the reconstructed particles uniformly randomly between $\pm$150 μm from its initial assigned location. For each detected (real) particle in each realization that help define the envelope of $g(r)$ values, the particle could have been placed in a 300 micron-wide swath centered on the previously reconstructed position. This, combined with the constant $\mathrm{d}r = 100\mu$m bin-width in computing the $g(r)$ curves used in this work, should help give a sense of how much uncertainty there is in $g(r)$ due to errors and biases resulting from artificially latching the z coordinates to a 100 μm resolution grid.

Instrument Sensitivity Nonuniformity. Although every effort was made to ensure that the domain of the holographic measurements were limited to regions of the reconstructed volume that were trustworthy, some of the signals that are being measured are quite small in magnitude and it can be difficult to convince a skeptical reader that the small deviations from perfect spatial randomness are not just due to subtle instrument sensitivity nonuniformities.

In the supplemental material for (3), this was addressed by comparing the measured RDF to an artificially "homogenized" data set that retains some elements of the variability of the natural dataset but with an RDF that deviates from unity only due to (i) number concentration fluctuations from hologram-to-hologram and (ii) instrument sensitivity nonuniformities. We made the "superhologram RDF" ($g_{sh}(r)$) using a similar approach here. Explicitly, each realization of a superhologram RDF was generated as follows:

- Create a "superhologram" of all particle detections within the flight interval (or subinterval). This "superhologram" is created by essentially taking the ensemble of all detected particle detection positions (x, y, z coordinates) no matter which hologram in the flight interval/subinterval they were associated with. (Essentially, superimpose the particle positions from each holographic volume into one holographic volume). This "superhologram" creates a list that has a total of N_{tot} particle positions, where $N_{\text{tot}} = \sum_i N_i$ and N_i is the number of detected particles in the ith hologram of the interval.
- Randomly permute the particle positions (shuffle the list) and then assign particles in the shuffled list numbered $1..N_1$ to the first reconstructed hologram, then place particles in positions between $N_1 + 1$ and $N_1 + N_2$ to the second reconstructed hologram, etc.
- For this "randomly permuted dataset", compute the RDF (including the "jitter" step described in the above section) to create an RDF for a "homogenized" (reshuffled) version of the flight interval.

Like in the actual data RDF plots, this process is repeated 100 times allowing for the creation of an envelope. Each member of this ensemble corresponds to an RDF where any departures from unity are due to some combination of (1) instrumental uncertainty/bias/nonuniformity, (2) sampling variability/non-homogeneity, (3) artificial effects from depth latching, and/or (4) real spatial clustering that persists for a significant fraction of the flight interval/subinterval.

This last point is somewhat subtle and deserves some additional commentary. Consider a system that truly has no spatial clustering, instrument imperfections, or drop number concentration fluctuations except for a small subset of consecutive holograms within the flight interval where there is real homogeneous droplet clustering. Because the droplets in the clustering regime form a small fraction of total particle detections, they will not have a large impact on the heat-map for the entire flight interval and $g_{sh}(r)$ for the entire flight interval would not deviate appreciably from unity. If, however, the small contiguous sub-interval where homogeneous droplet clustering was the entire domain that was explored, this may significantly impact the heat-map (superhologram) for the sub-interval. For intervals only a few holograms long, the superhologram may have incorporated real, transient, localized spatial clustering as a non-uniform instrument detection heat-map. Because the superhologram may only be created from a few holograms and the number of both "measured" and "expected in perfect randomness" particle pairs separated by $(r, r + \mathrm{d}r)$ is small, then the measured estimate of $g(r)$ becomes increasingly uncertain and – following the analysis presented in detail in (3) – the uncertainty envelope and median values for $g_{sh}(r)$ increase significantly.

This situation is not of mere academic interest, but rather explains why the bottom row of the clustering "classification" map ends up completely in the "insufficient" categorization (see Figure 4 in main paper). There likely is significant spatial clustering in the 2-hologram intervals immediately below the 4-hologram sub-section marked with a star, but – since the superhologram here is just combining two (clustered) holograms – the difference between the "data" and "shuffled" datasets aren't that different and there is insufficient evidence for there to be a statistical difference between $g(r)$ and $g_{sh}(r)$. Both curves move with $g(r)$ in significant excess of unity (suggesting clustering), but our tool to distinguish "unambiguous clustering"

from "clustering that could be spuriously induced/inferred" no longer has the necessary fidelity to determine this for certain. This is why we don't want to argue that "insufficient" regions means "no clustering". We believe the tools we have developed here are unlikely to give a false positive (if something is colored green, we are confident there is evidence for spatial clustering somewhere within that domain, hence the "unambiguous" label), but we believe the tool we have developed may give false negatives with some regularity, especially for small sub-intervals containing only a few consecutive holograms.

D. Further examples of clouds that exhibit clustering hotspots. As mentioned in the main manuscript, the finding of localized clustering hotspots is not unique to the section shown in Figure 4 of the main manuscript. In fact, in the holographic data from the MPCK$^+$ of the EUREC^{4}A campaign, droplet clustering is observed either in localized hotspots or, in some cloud transects, not at all. In Figure S1 we present the clustering evidence results for two sections of similar length that highlight the presence of clustering hotspots in different clouds on the same day (left) and clouds measured on a different day (5 days after the section shown in the main manuscript).

E. Additional Tests: Investigating possible influence of holographic arm on RDF. The holographic sample volume is situated between two "arms" of the MPCK$^+$ instrument: the upper arm, which houses the camera optics, and the lower arm, which contains the laser optics (7). The distance between the two arm windows is 21.9 cm. As discussed earlier, we selected a $1.1\,\text{cm} \times 1.1\,\text{cm} \times 6\,\text{cm}$ sub-volume within the MPCK$^+$ system. This sample volume is positioned 4 cm from the upper arm window and 11.9 cm from the lower arm window, corresponding to reconstructed z-positions from 6 to 12 cm. The upper z-limit of 12 cm was determined by the measurement system's limited depth resolution, while aggregated heatmaps of detected particles (across multiple holograms) confirm that setting the lower z-limit at 6 cm from the image plane ensures that any influence from the top (low z) arm on the airflow and droplet distribution is negligible.

The depth resolution of MPCK$^+$ is discussed in detail elsewhere (8). As an independent check on the influence of the top arm on the small z-position part of the sample volume (in addition to the heatmap analysis), we reanalyzed the 55 m holographic segment presented in the main paper, restricting the reconstructed z-positions to 9–12 cm such that the sample volume is at least 7 cm away from the windows of both arms, and influences should be minimal. The second row of Figure S2 shows the results of this reduced-volume analysis in the same format used in the main paper for localized RDF signatures.

Overall, the results closely match those obtained using the larger volume. While the differences between the top two rows of the figure can be attributed to fewer sampled droplets (and thus greater uncertainty), the primary features remain consistent. In particular, clear evidence of small-scale clustering persists, although the reduced sample size (approximately half) leads to certain "unambiguous" sections being reclassified as "possible." Nonetheless, the opacity values indicate that the median separation of the envelopes remains virtually the same; only envelope thickness increases due to the lower sample count. We therefore conclude that our initial choice of sample volume was valid, and that the observed, highly localized clustering signatures are unaffected by potential influences from the holographic arms.

F. Additional Tests: Choice of Minimum Diameter in RDF calculations. To investigate the influence of restricting the droplet size to >10 μm in the main analysis on the clustering hotspots, we recalculated the RDF for 16-hologram sub-sections of the cloud section shown in the main manuscript, applying different droplet size restrictions. The results are presented in Figure S2, using the same color scheme as in Figure 4 of the main text. The top row shows the original results for retaining all detected drops >10 μm, while the third to eighth rows present results for different minimum and maximum droplet diameters d. Since the droplet size distribution in the entire analyzed cloud section is relatively narrow (see Figure 1 in the main manuscript), further restricting the droplet sizes reduces the number of droplets included in the RDF calculation. As a result, some sub-sections, originally showing "unambiguous" clustering evidence, are classified as showing "insufficient" or "possible" clustering evidence, despite exhibiting a strong signal S as indicated by the opacity of the colors. Qualitatively, however, the sub-sections identified as clustered, or those showing a strong signal, are consistent across all applied droplet size restrictions. When analyzing only droplets > 16 μm, a few additional sub-sections exhibit a strong signal. However, due to the very limited number of droplets in these cases, the clustering evidence is still classified as "insufficient", making it unclear whether these are true additional clustered regions or merely statistical artifacts.

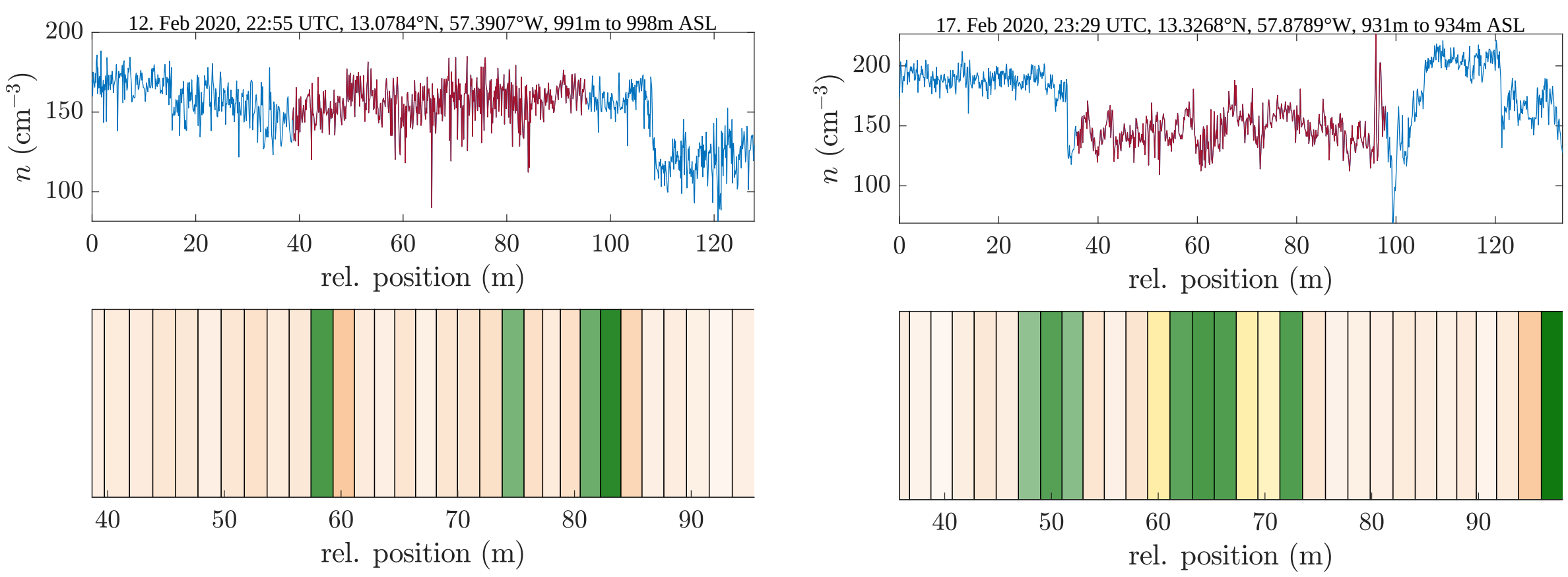

Fig. S1. Top panels show a number concentration profile (determined from holographic measurements) along ∼130 m horizontal transects of two clouds. The date, time, location and altitude of the transects can be found on top of the graphs. The RDF of the section shown in red is calculated based on 16-hologram sub-sections and the results are each shown on the bottom with the same color scheme as used in Figure 4 of the main manuscript.

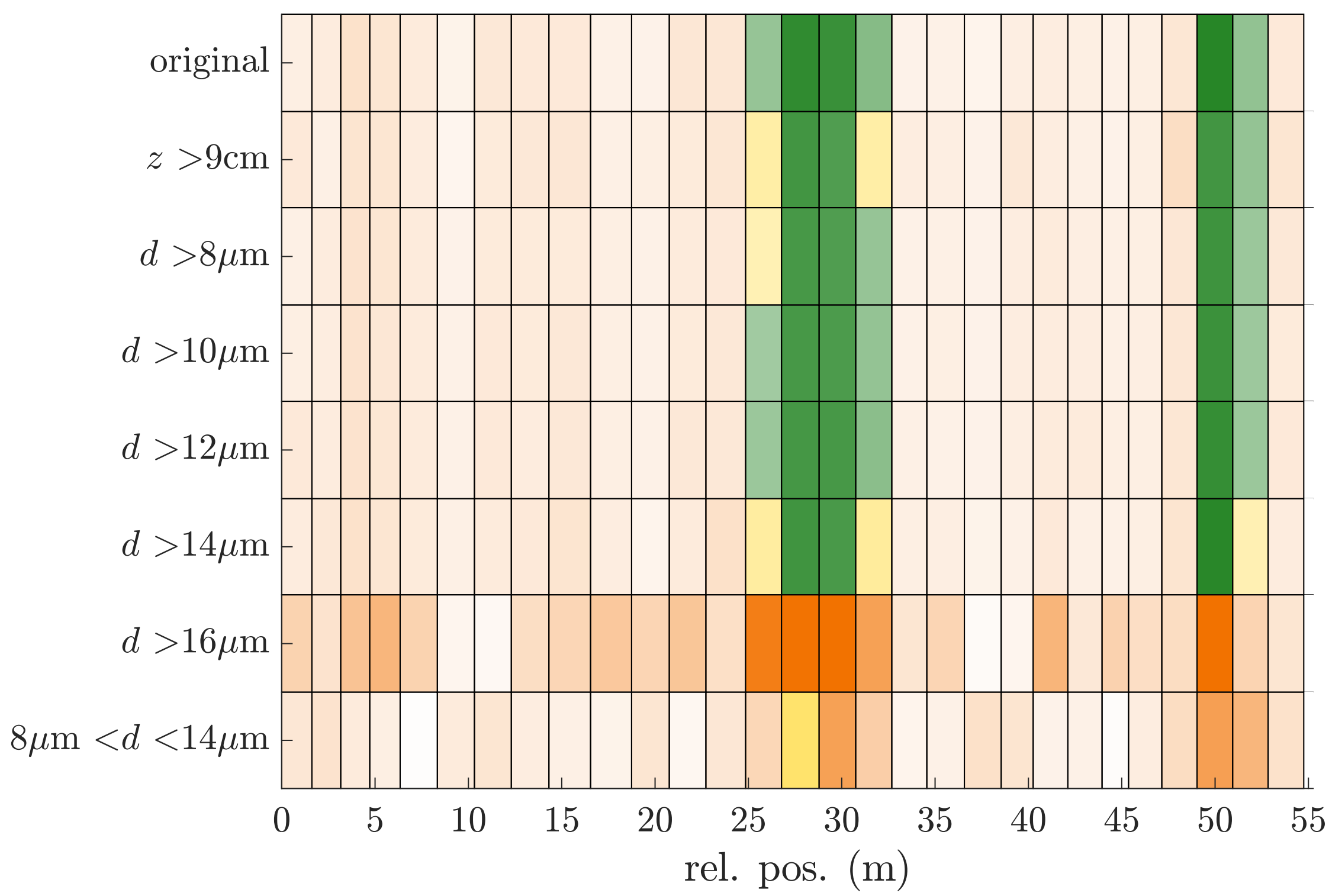

Fig. S2. The clustering charts for the analyzed 55 m cloud segment. The color indicates the clustering group ("unambiguous," "possible," or "insufficient"), while the opacity reflects the difference $S\left(g - g_{\mathrm{sh}}\right)$ between $g(r)$ and $g_{\mathrm{sh}}(r)$. The top row presents results for the 16-hologram sub-sections, as illustrated in Figure 4 of the main text (4 cm away from the upper window and 11.9 cm from the lower window, minimum droplet diameter 10 μm). The second row displays the corresponding analyses of these same sub-sections using a reduced probing volume (at least 7 cm away from either windows) for the RDF calculations. The following rows show the clustering evidence when restricting the analyzed droplets to different size ranges. The changes from clustering evidence group can be explained due to lower number of droplet pairs, when restricting droplet size and hence less statistics such that an "unambiguous" signal is not reached anymore.

References

1. M Larsen, R Shaw, A method for computing the three-dimensional radial distribution function of cloud particles from holographic images. *Atmospheric Meas. Tech.* **11**, 4261–4272 (2018).
2. D Stoyan, W Kendall, J Mecke, *Stochastic Geometry and its Applications.* (Wiley, Chichister, England), (1995).
3. M Larsen, R Shaw, A Kostinski, S Glienke, Fine-scale droplet clustering in atmospheric clouds: 3d radial distribution function from airborne digital holography. *Phys. Rev. Lett.* **121**, 204501 (2018).
4. W Newman, J Lew, G Siscoe, R Fovell, Systematic efects of randomness in radiative transfer. *J. Atmospheric Sci.* **52**, 427–435 (1995).
5. A Kostinski, On the extinction of radiation by a homogeneous but spatially correlated random medium. *J. Opt. Soc. Am. A* **18**, 1929–1933 (2001).
6. M Larsen, Ph.D. thesis (Michigan Technological University) (2006).
7. G Bagheri, et al., The max planck cloudkite (mpck): High-resolution airborne measurements of turbulence and cloud microphysics. *EGUsphere* **2026**, 1–44 (2026).
8. B Thiede, et al., In-line holographic droplet imaging: accelerated classification with convolutional neural networks and quantitative experimental validation. *Atmospheric Meas. Tech.* **18**, 6291–6314 (2025).
